# Multiple Breath Washout for Early Assessment of Pulmonary Complications in Patients With Primary Antibody Deficiencies: An Observational Study in Pediatric Age

**DOI:** 10.3389/fped.2022.773751

**Published:** 2022-05-17

**Authors:** Teresa Secchi, Lucia Augusta Baselli, Maria Chiara Russo, Irene Maria Borzani, Federica Carta, Maria Amalia Lopopolo, Michaela Foà, Adriano La Vecchia, Carlo Agostoni, Massimo Agosti, Rosa Maria Dellepiane

**Affiliations:** ^1^University of Milan, Milan, Italy; ^2^Pediatric Intermediate Care Unit, Fondazione IRCCS Cà Granda Ospedale Maggiore Policlinico, Milan, Italy; ^3^Cystic Fibrosis Regional Reference Centre, Fondazione IRCCS Ca' Granda Ospedale Maggiore Policlinico, Milan, Italy; ^4^Radiology Unit-Pediatric Division, Fondazione IRCCS Ca' Granda Ospedale Maggiore Policlinico, Milan, Italy; ^5^Pediatric Rehabilitation Unit, Fondazione IRCCS Ca' Granda Ospedale Maggiore Policlinico, Milan, Italy; ^6^Department of Clinical Sciences and Community Health (DISCCO), University of Milan, Milan, Italy; ^7^Woman and Child Department, Ospedale “Filippo Del Ponte,” University of Insubria, Varese, Italy

**Keywords:** human inborn errors of immunity, primary antibody deficiencies, lung clearance index, multiple breath washout, spirometry, high resolution computed tomography, pulmonary complications, respiratory function

## Abstract

**Background:**

In primary antibody deficiencies (PADs), pulmonary complications are the main cause of morbidity, despite immunoglobulin substitutive therapy, antibiotic treatment of exacerbations, and respiratory physiotherapy. Current Italian recommendations for surveillance of PADs respiratory complications include an annual assessment of spirometry and execution of chest high-resolution computed tomography (HRCT) every 4 years.

**Objective:**

This study aimed to evaluate the effectiveness of the lung clearance index (LCI) as an early marker of lung damage in patients with PADs. LCI is measured by multiple breath washout (MBW), a non-invasive and highly specific test widely used in patients with cystic fibrosis (CF).

**Methods:**

Pediatric patients with PADs (*n* = 17, 10 male, 7 female, and age range 5–15 years) underwent baseline assessment of lung involvement with chest HRCT, spirometry, and multiple breath nitrogen washout. Among them, 13 patients were followed up to repeat HRCT after 4 years, while performing pulmonary function tests annually. Their baseline and follow-up LCI and forced expiratory volume at 1 s (FEV1) values were compared, taking HRCT as the gold standard, using logistic regression analysis.

**Results:**

Lung clearance index [odds ratio (OR) 2.3 (confidence interval (CI) 0.1–52) at baseline, OR 3.9 (CI 0.2–191) at follow-up] has a stronger discriminating power between altered and normal HRCT rather than FEV1 [OR 0.6 (CI 0.2–2) at baseline, OR 1.6 (CI 0.1–13.6) at follow-up].

**Conclusion:**

Within the context of a limited sample size, LCI seems to be more predictive of HRCT alterations than FEV1 and more sensitive than HRCT in detecting non-uniform ventilation in the absence of bronchiectasis. A study of a larger cohort of pediatric patients followed longitudinally in adulthood is needed to challenge these findings.

## Introduction

Primary antibody deficiencies (PADs), a heterogeneous spectrum of conditions characterized by the marked reduction in blood levels of at least one main immunoglobulin (Ig) class, are the most representative diseases among all human inborn errors of immunity, accounting for about half of the cases of primary immunodeficiency (1–6).

Pulmonary diseases, especially respiratory tract infections, are among the main causes of morbidity and mortality in patients with PADs, although their prognosis has been significantly improved by adequate immunoglobulin substitutive therapy, aggressive antibiotic therapy for sinopulmonary exacerbations, and personalized respiratory physiotherapy programs. The presence of chronic lung injury at diagnosis represents the major predictor of mortality, while an early diagnosis and a timely start of Ig substitutive therapy, together with well-conducted respiratory physiotherapy, are predictors of positive outcomes for patients with PADs (7–11).

Respiratory tract infections can be relevant when occurring acutely, but their recurrence can also have long-term effects on the lung architecture by remodeling airways. As a result of repeated infectious and inflammatory episodes, bronchiectasis is common in patients with PADs. Chest high-resolution computed tomography (HRCT) is currently the gold standard for the detection and characterization of bronchiectasis and respiratory complications (10, 12).

There are no international consensus guidelines on monitoring lung diseases in patients with PADs, resulting in great variability between centers (9). The current Italian Association of Pediatric Hematology and Oncology (AIEOP)/Italian Primary Immunodeficiencies Network (IPINet) recommendations for the surveillance of respiratory complications in PADs recommend the annual evaluation of lung function with spirometry, associated with chest HRCT every 4 years in otherwise clinically stable patients. Spirometry has to be carried out in wellbeing or at least 3 weeks after recovery from an episode of respiratory exacerbation, while HRCT must be anticipated in case of an episode of severe respiratory exacerbation requiring hospitalization or if the patient is not clinically healthy and possibly performed at least 3 weeks after recovery (13).

Spirometry detects dynamic lung volumes by measuring the movement of the inhaled and exhaled air during respiratory maneuvers and by expressing them as a function of time. One of the parameters detected during spirometry is the forced expiratory volume at 1 s (FEV1), the volume of air exhaled in the first second of a forced exhalation. FEV1 represents the index parameter of the bronchial obstruction degree, as well as being a good indicator of airway resistance. FEV1 is commonly used as the primary tool in monitoring patients with bronchiectasis (8, 9, 14). However, both in literature and in clinical practice, there is increasing evidence that FEV1 is poorly sensitive in detecting early lung damage. Most clinical trials performed on patients with bronchiectasis showed that the initial pulmonary impairment identifiable through chest HRCT was not detectable by FEV1, which was normal in most patients (15–18). On the other hand, exposing patients with PADs to radiation increases the risk of neoplasms in the long term. Due to the underlying disease, patients with PADs have a greater risk of neoplastic disease compared with the general population. Minimizing radiation exposure in pediatric patients with PADs is mandatory as well as considering the improvement in their life expectancy over the past years (19).

Multiple breath inert gas washout (MBW) assesses static lung volumes and inhomogeneity of lung ventilation during tidal breathing providing, as a functional parameter, the lung clearance index (LCI), the number of lung volumes required to complete the washout of a tracer gas inhaled or already present in the lungs. For the execution of this non-invasive respiratory test, only minimal cooperation is required from the patient, so that it is potentially applicable to all ages. MBW has therefore assumed increasing clinical importance in the follow-up of chronic pulmonary disease in pediatric patients, such as those with cystic fibrosis (CF) (20–22).

On the whole, observations in patients with CF suggest that the MBW determination may provide a repeatable and highly sensitive surveillance method for monitoring lung diseases in patients with bronchiectasis, particularly those with preserved spirometry (23–25).

This study aimed to evaluate the effectiveness of LCI, measured by MBW, as an early marker of lung damage and to evaluate its trend over time during the follow-up of respiratory complications in patients with PADs.

## Methods

### Trial Design and Participants

A 4-year non-profit single-center prospective observational design has been conducted from December 2015 to January 2020. The study was approved by the Ethics Committee (EC) of the IRCCS Fondazione Ca' Granda (EC approval number: 55_2018bis).

The primary goal of the study was to evaluate the effectiveness of LCI, measured by nitrogen MBW, as an early marker of lung damage in patients with PADs, even in the absence of bronchiectasis. The secondary goal of the study was to evaluate the overtime trend of LCI in patients with PADs during follow-up of respiratory complications and to study the correlation between MBW and HRCT alterations.

Inclusion criteria in the study were: patients suffering from PADs diagnosed according to the European Society for Immunodeficiencies (ESID) criteria (26); patients in the follow-up of the respiratory complications according to the AIEOP/IPINet recommendations (13); availability of chest HRCT performed in wellbeing at least once in the last 4 years; age > 5 years; compliance with the study protocol and ability in performing pulmonary function tests (PFTs) according to the criteria of reliability. Exclusion criteria were considered age < 5 years; patients not cooperating and/or not able to carry out the test correctly.

### Intervention

According to the study design, MBW with LCI determination has been included in the standard follow-up of patients with PADs to monitor the progression of respiratory complications. At the time of enrollment, each patient has been subjected to a global baseline assessment of lung involvement by performing spirometry, nitrogen MBW, and chest HRCT. For 13 out of the 17 patients with stable conditions, the PFTs (spirometry and MBW), performed annually, have been compared with chest HRCT, performed every 4 years. For the other 4 patients, enrolled later, the follow-up was too limited to check repeated chest HRCT and comparison has been possible only at baseline time.

High-resolution computed tomography scan studies were scored using the Bhalla score (27) in random order by a pediatric radiologist and a pediatric pulmonologist, blinded to patients' identities and clinical information, respectively. Readers evaluated each examination independently and reported in consensus (28, 29).

The Bhalla score (27) is a morphological scoring system usually applied to monitor pulmonary disease progression and stage of chronic broncho-pneumopathies. The Bhalla score can vary between 0 and 25 and includes the presence, severity, and extent of different pulmonary alterations, such as bronchiectasis, peri-bronchial thickening, mucous plugging, sacculation/abscesses, bullae, emphysema, and collapse/consolidation (12, 30).

Each MBW test consists of a wash-in phase and a wash-out phase. This respiratory test is feasible in two ways using an extrinsic inert tracer gas, inhaled until equilibrium is reached and then eliminated by breathing ambient air, or by inhaling 100% oxygen for the washout of nitrogen, an intrinsic inert gas normally present in the airways. In our survey, 100% oxygen was used for the nitrogen washout to increase sensitivity in identifying abnormalities, nitrogen being present in all areas of the lungs (20, 21).

Parameters derived from MBW are obtained through the analysis of the breath-by-breath concentrations and volumes of gas. The LCI may be considered the most reliable MBW parameter since it reflects the inhomogeneity of pulmonary ventilation and is defined as the number of lung turnovers required to washout the tracer gas up to 2.5% of its initial concentration. In a healthy person, this takes about 5–7 turnovers. According to a 2011 review by Fuchs and Gappa (15), which reports the most relevant studies conducted to define LCI normal values and Upper Limit of Normal (ULN) in pediatric age, LCI is constant during childhood and independent of age, weight, height, and gender during adolescence. The value of 7.00 is reported as ULN for LCI in childhood. In the case of non-uniform ventilation, LCI increases with the number of turnovers needed for the tracer gas to be eliminated from the lungs (15, 20, 22, 31).

### Statistical Methods

Parameters of the respiratory function tests considered for the study were FEV1 as a percentage of the predicted value (FEV1 %predicted) and LCI at 2.5% of the gas initial concentration (LCI 2.5%) calculated with nitrogen MBW.

Regarding the cross-sectional evaluation, LCI and FEV1 parameters were compared, taking the HRCT as the gold standard, evaluated by the Bhalla score. Given the non-normal and asymmetrical distribution of the Bhalla score, to better analyze the data, HRCT was transformed into a dichotomous variable, and statistical analyses were accordingly performed by splitting the patients into two groups. Accordingly, HRCT has been considered normal (N-CT) for patients with Bhalla score = 0, while it has been considered altered (A-CT) for patients with Bhalla score > 0. Fisher's exact test has been employed to test the dependency on gender, while age, FEV1, and LCI were compared using the Wilcoxon–Mann–Whitney test. Univariate and multivariate (adjusting for gender and age) logistic regression analyses were used to study the association between FEV1, LCI, N-CT/A-CT, and Bhalla scores 0–2 or more than 2. We calculated the 95% confidence interval (*CI*) using the bootstrap method.

## Results

In this study, 17 patients (10 male and 7 female, age range between 5.7 and 14.8 years) with PADs were involved, the median age at baseline was 10.7 years [interquartile range (IQR) 8.5–12.6], while the median age at the HRCT follow-up after 4 years was 14.8 years (IQR 13.7–16.5).

Regarding the PADs, 11 patients (6 male and 5 female) were diagnosed with Common Variable Immunodeficiency (CVID), 2 male patients with X-linked agammaglobulinemia (XLA), and 1 male patient with autosomal recessive agammaglobulinemia (ARA), 2 female patients with selective IgA deficiency (SIgAD) with respiratory complications and 1 male with hyper IgM syndrome (HIGM). Furthermore, 12 out of the 17 patients (8 CVID, 2 XLA, 1 ARA, and 1 HIGM) were undergoing Ig substitutive therapy at the time of the study, all with adequate pre-infusion IgG levels (median 915 mg/dl and IQR 872–964). All patients were also following a personalized respiratory physiotherapy program. The median age of symptoms onset in all patients involved was 1.5 years (IQR 1.0–3.3). The median age at diagnosis was 6.6 years (IQR 4.3–9.8). The median diagnosis delay was 3.2 years (IQR 1.3–6.7). The median duration of follow-up for this study was 4.0 years (IQR 3.2–4.0) (Table 1).

**Table 1:** Clinical and demographic characteristics of the population in the study.

**Variable**	***N* (%)**	**Mean (SD)**	**Median (IQR)**	**Range**
Age of symptoms onset (years)	17 (100%)	2.7 (3.1)	1.5 (1.0–3.3)	0.0–11.6
Age at diagnosis (years)	17 (100%)	7.0 (4.2)	6.6 (4.3–9.8)	0.7–14.8
Diagnosis delay (years)	17 (100%)	4.3 (3.8)	3.2 (1.3–6.7)	0.4–11.9
Age at baseline HRCT (years)	17 (100%)	10.4 (3.0)	10.7 (8.5–12.6)	5.7–14.8
Age at HRCT follow-up (years)	13 (76%)	14.7 (2.8)	14.8 (13.7–16.5)	9.1–18.1
Duration of follow-up (years)	17 (100%)	3.4 (1.2)	4.0 (3.2–4.0)	0.4–4.9
Pre-infusion IgG levels (mg/dl)	12 (71%)	910 (107)	915 (872–964)	716–1,080

*IgG, immunoglobulin G; IQR, Interquartile range; SD, Standard deviation*.

**Figure 1:**
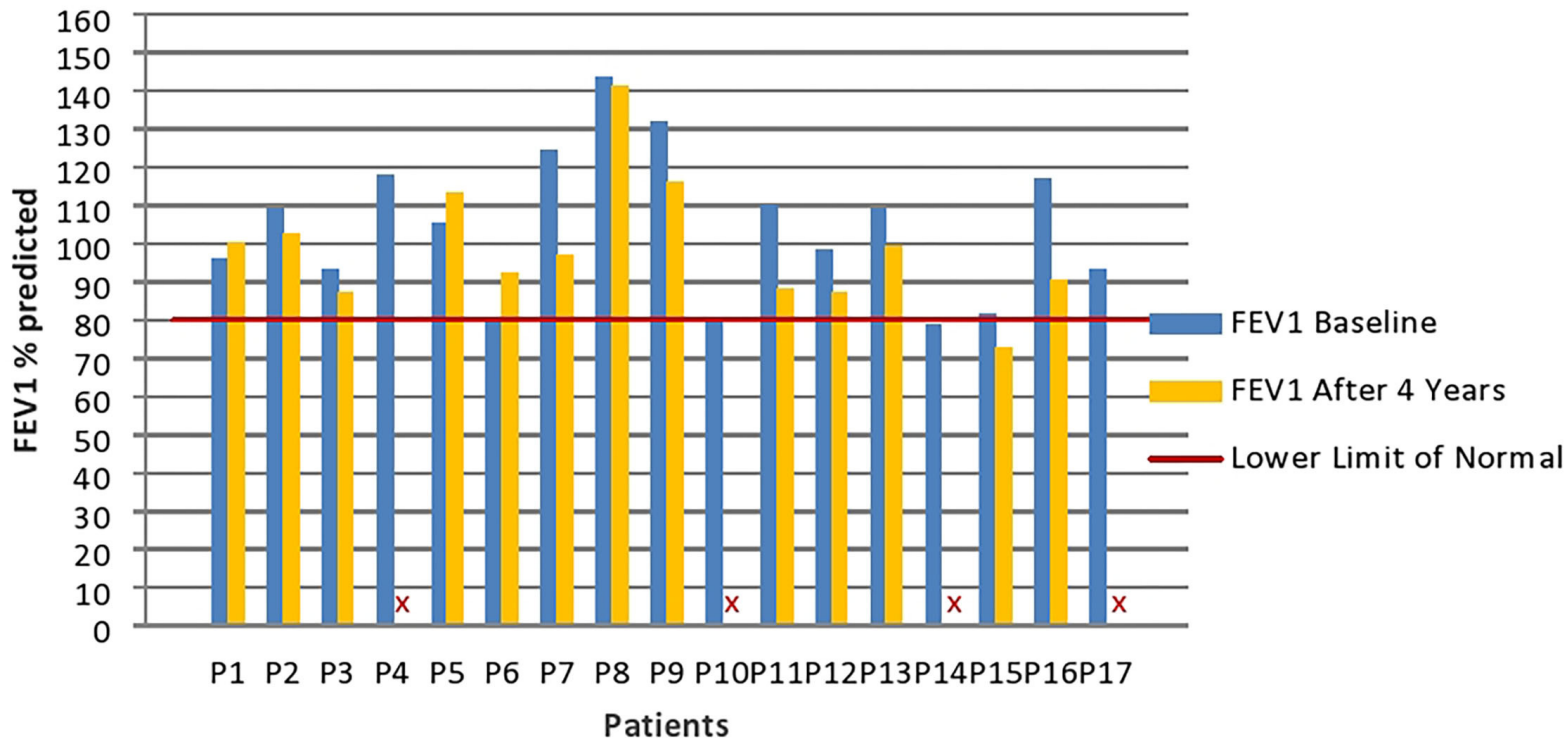
Patients' FEV1 values (percentages values compared with the predicted) at baseline and high-resolution computed tomography (HRCT) follow-up. Four patients (P4; P10; P14; and P17) did not perform spirometry at follow-up. FEV1 %predicted, Forced Expiratory Volume at 1 s percentage values compared to the predicted.

Spirometry turned out to be normal or slightly altered in all enrolled patients. FEV1 below 80% of the predicted values, considered as Lower Limit of Normal (LLN) (14), has been found 4 times out of 56 measurements (7.14% of cases). In two of these cases, spirometry was repeated in otherwise stable patients 3 months later, following an intensification of the respiratory physiotherapy program, with a return to normal basal values. The median FEV1 value observed at the baseline time was 105.2% (IQR 93.1–117.1) and 97.2% (IQR 88.0–102.4) at the time of the second HRCT (Figure 1).

**Figure 2:**
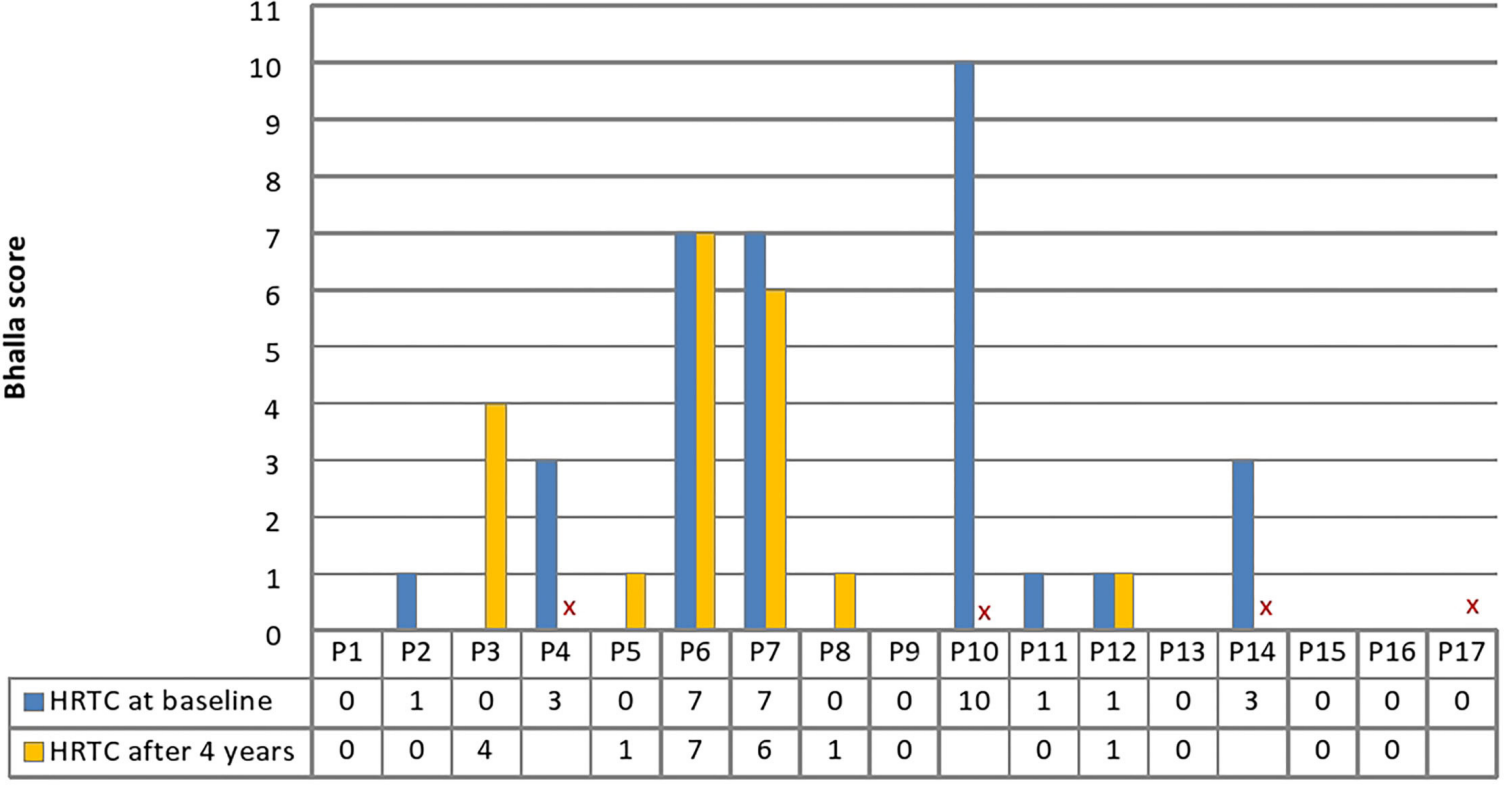
Patients' Bhalla score at baseline and HRCT follow-up. A Bhalla score of 0 is normal, 0–4 is indicative of mild alterations, and >4 is indicative of moderate or severe alterations. Four patients (P4; P10; P14; and P17) did not perform HRTC at follow-up. HRCT, high resolution computed tomography.

Although spirometry was always normal or just mildly altered, HRCT was found altered in 8/17 (47%) patients at baseline, and in 6/13 (46%) patients at follow-up after 4 years. At baseline, 3 patients with A-TC (18%) were reported with moderately altered Bhalla scores (median Bhalla score 7.0, IQR 7.0–8.5) and 5 patients with A-TC (29%) with slightly altered Bhalla scores (median Bhalla score 1.0, IQR 1.0–3.0). At the time of follow-up, 2 patients with A-TC (15%) were reported with moderately altered Bhalla scores (median Bhalla score 6.5, IQR 6.25–6.75) and 4 patients with A-TC (31%) with slightly altered Bhalla scores (median Bhalla score 1.0, IQR 1.0–1.75) (Figure 2). The HRCT images of patients with moderately altered Bhalla scores showed the presence of bronchiectasis with thickened walls and other pathological findings, such as collapse and consolidation, peri-bronchial thickening, and mucus plugs. Regardless of the severity and extent of bronchiectasis, both of which were mild in 2 out of 3 patients, the severity of the pulmonary picture is determined by the involvement of the more distal bronchioles.

**Figure 3:**
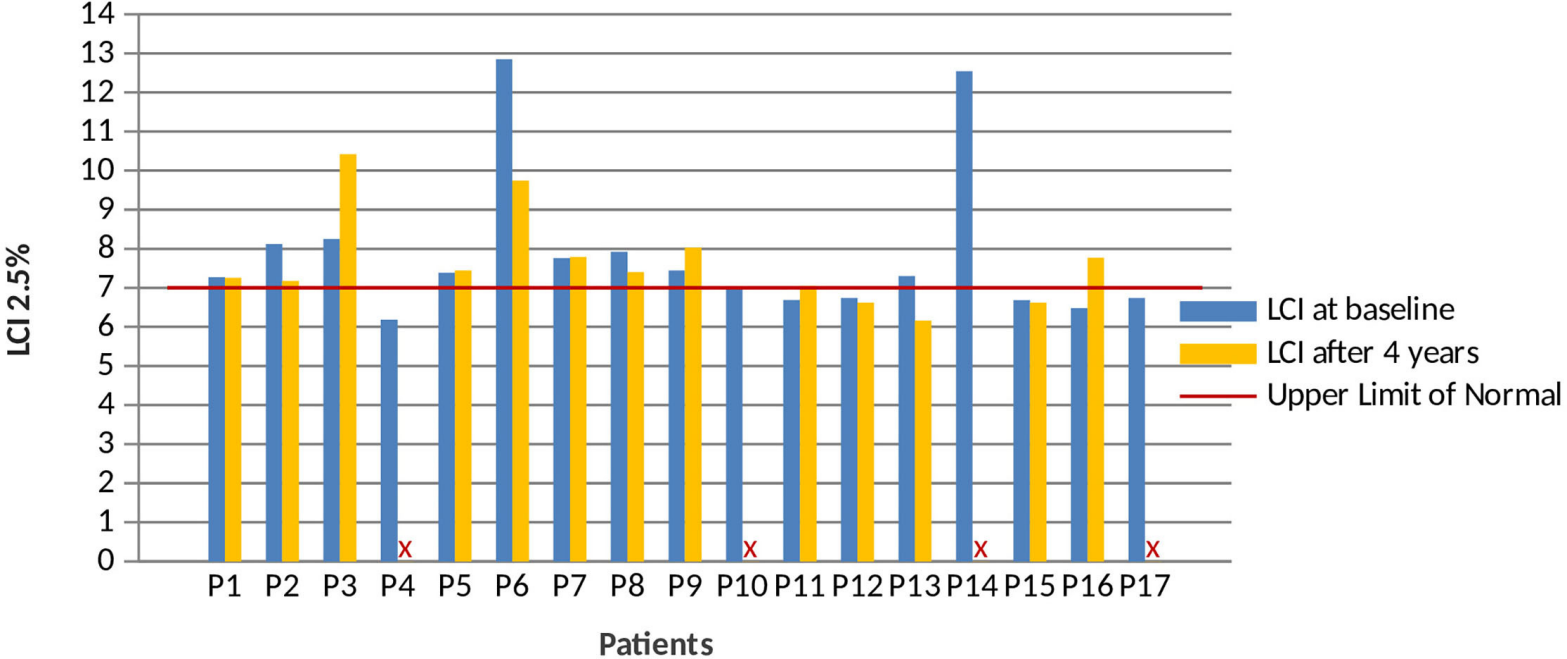
Patients' LCI at 2.5% of gas initial concentration at baseline and HRCT follow-up. Four patients (P4; P10; P14; and P17) did not perform multiple breath washout (MBW) at follow-up. LCI 2.5%, lung clearance index at 2.5% of the gas initial concentration.

The lung function study was completed through the evaluation of ventilatory inhomogeneity by nitrogen MBW with LCI detection at 2.5% of the initial concentration of tracer gas. LCI values > 7 were found in more than 50% of patients. The median LCI value at the baseline time was 7.3 (IQR 6.74–7.92) and 7.4 (IQR 7.05–7.79) at the time of follow-up corresponding to the second HRCT (Figure 3).

**Figure 4:**
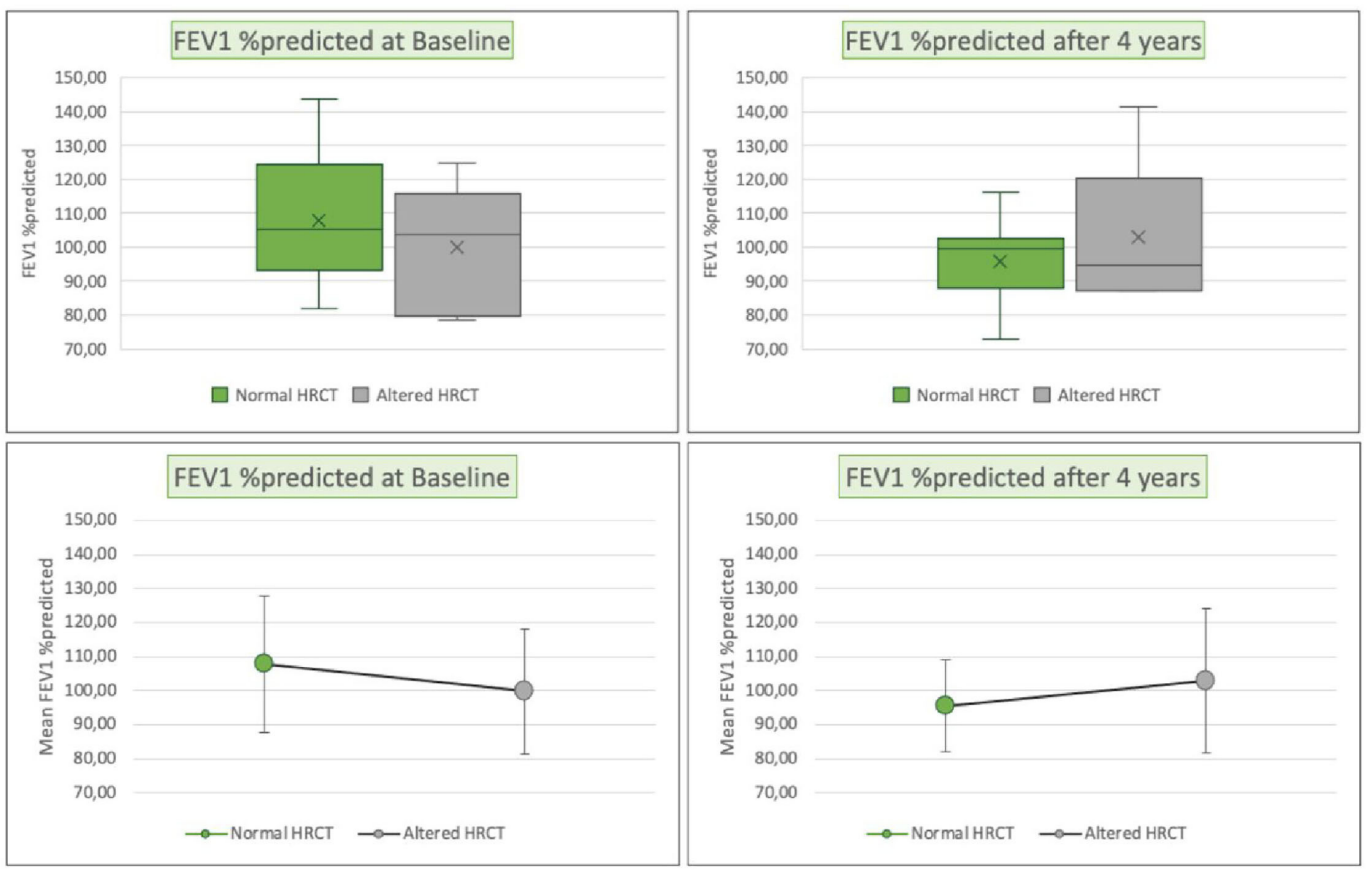
Box plots (above), mean and standard deviation (SD) (below) of FEV1 (percentages values compared with the predicted), respectively, for patients with normal HRCT (green) or altered HRCT (gray), at enrollment (left), and at the HRCT follow-up (right). FEV1 %predicted, Forced Expiratory Volume at 1 s percentage values compared to the predicted; HRCT, high resolution computed tomography.

**Figure 5:**
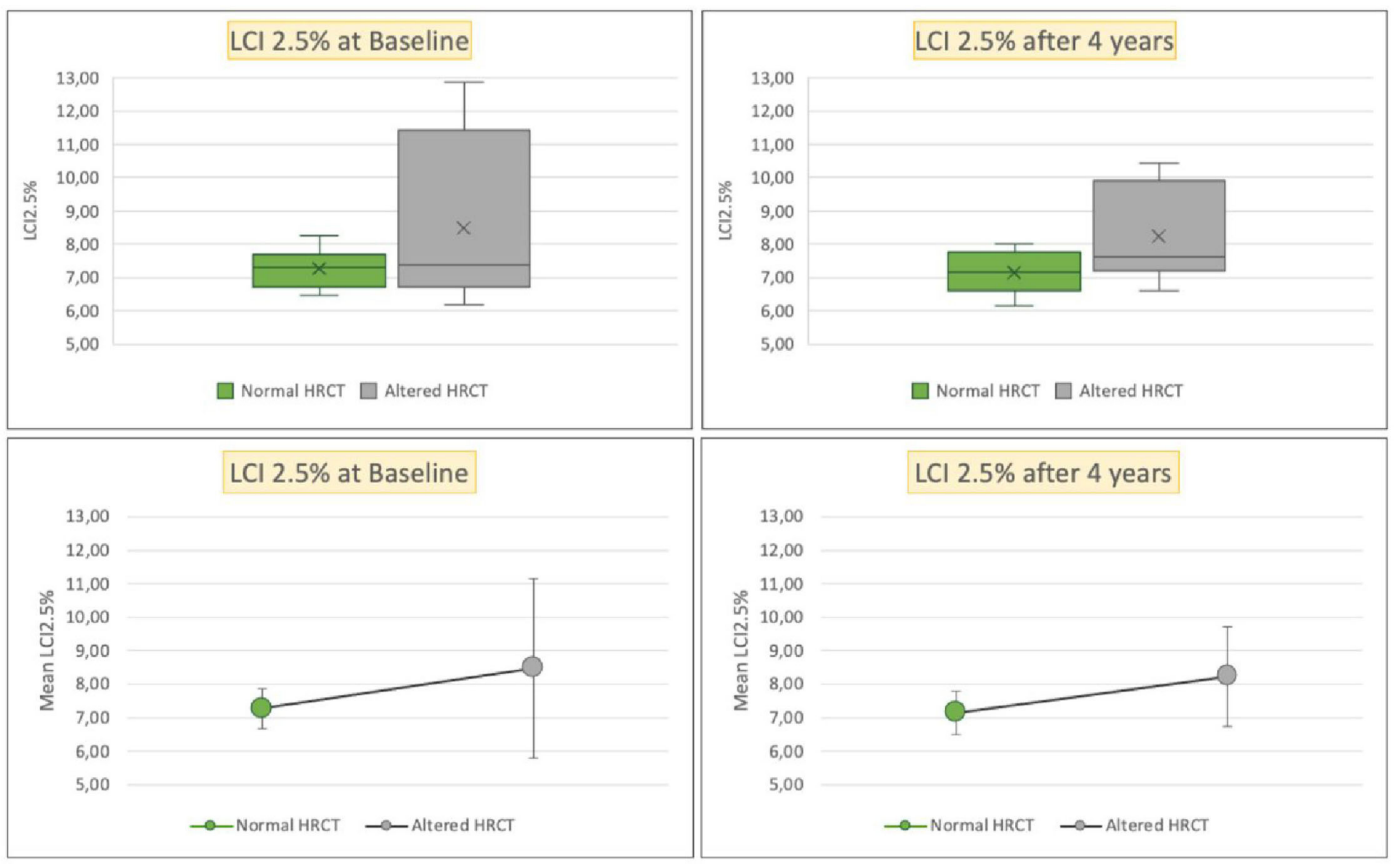
Box plots (above), mean and SD (below) of LCI at 2.5% of gas initial concentration, respectively, for patients with normal HRCT (green) or altered HRCT (gray), at enrollment (left), and at the HRCT follow-up (right). HRCT, high resolution computed tomography; LCI 2.5%, lung clearance index at 2.5% of gas initial concentration.

**Figure 6:**
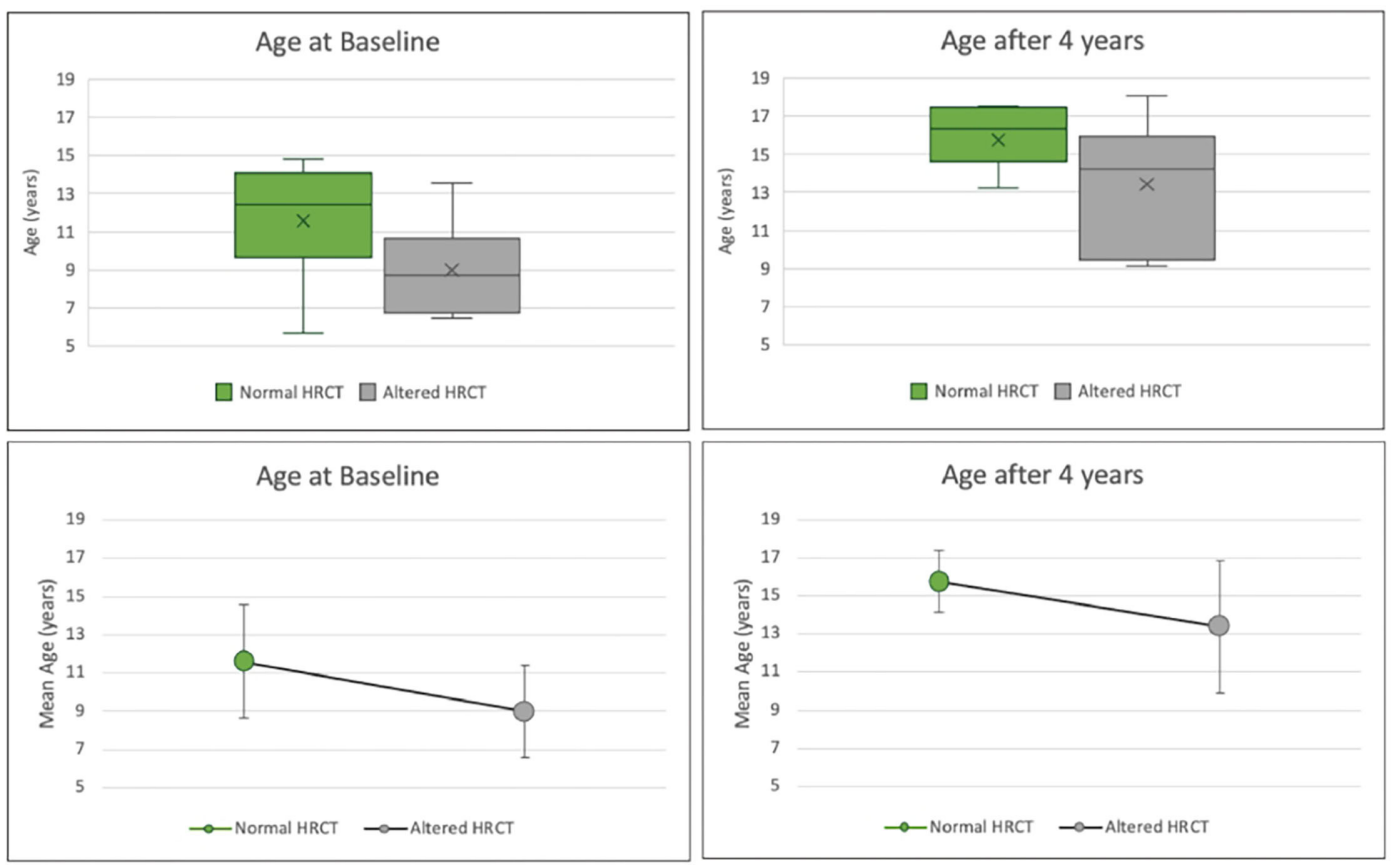
Box plots (above), mean and SD (below) of the age in years, respectively, for patients with normal HRCT (green) or altered HRCT (gray) at enrollment (left) and at the HRCT follow-up (right). HRCT, high resolution computed tomography.

At baseline and at the time of radiological follow-up, LCI and FEV1 parameters were compared taking HRCT as the gold standard. No significant differences emerged in terms of the distributions of FEV1 and LCI between patients with normal or altered HRCT (Figures 4, 5). The comparison of ages of the two groups of patients was examined by the Wilcoxon–Mann–Whitney test (*p* = 0.04) with younger patients showing a more altered HRCT (Figure 6).

After standardizing FEV1 and LCI values, univariate logistic regression analyses were performed, using N-CT/A-CT as the dependent variable. FEV1 at baseline showed a weak negative association with A-CT (OR = 0.6, CI 0.2–2, *p* = 0,4). On the contrary, LCI at baseline showed a positive association (OR = 2.3, CI 0.1–52, *p* = 0.4). The same analyses were performed with the sample of 13 patients during the 4 years after HRTC follow-up. After 4 years, FEV1 showed a weak positive association with A-CT (OR = 1.6, CI 0.1–13.6, *p* = 0,4), while LCI showed a stronger positive association after 4 years (OR = 3,9, CI 0.2–191, *p* = 0.2). We found a stronger positive correlation of LCI than FEV1 with structural lung abnormalities at HRCT even when we performed multivariate (age and gender-adjusted) logistic regression analysis and when we changed the dependent variable in the Bhalla scores 0–2 or more than 2 (Supplementary Table 1).

## Discussion

Multiple breath washout is sensitive to minor changes at an early stage of lung disease and less dependent on the cooperation of the patient (32). Studies on CF (33), primary ciliary dyskinesia (34), and non-CF bronchiectasis (35) demonstrated a correlation between abnormal LCI and structural lung disease. A recent study has demonstrated the superiority of MBW compared with spirometry in detecting pulmonary dysfunction in long-term survivors of childhood cancer (36). MBW is a promising method for the assessment of the lungs in PADs (32).

To our knowledge, this is the first study evaluating LCI in the pediatric population of patients with inborn errors of immunity. Our observations suggest that LCI might be a parameter with higher sensitivity when compared with FEV1 in detecting lung abnormalities, even in clinically stable situations. Except in one single case, the PFTs were always performed in stable conditions, and no patient had experienced significant respiratory exacerbations before performing the MBW.

Although all patients presented with various degrees of airway disease, 82% of patients with altered HRCT at baseline and/or follow-up had FEV1 values within the normal range, despite their LCI values being significantly altered in 64% of cases. Among patients with normal HRCT at baseline or follow-up, 91% had FEV1 values within the normal range, but 82% showed slightly altered LCI values. Nevertheless, alterations in LCI found in cases of normal pulmonary HRCT were milder than those found in patients with impaired chest HRCT.

Alterations in very small airways can result in ventilation inhomogeneity. Since HRCT could not detect these alterations, LCI may be more sensitive to initial lung involvement than HRCT in patients with CF (32). The authors believe that validating MBW in patients with PADs could change their clinical management.

A major limitation of this study is the small sample size, limiting the power of the study. Furthermore, the patients suffer from a heterogeneous group of PADs. Therefore, our results should be interpreted with care. Nevertheless, the results from our monocentric setting suggest the need to check in larger groups the higher predictive value of an increase in LCI, compared with a reduction in FEV1, in evaluating alterations in pulmonary HRCT. We find a significant difference in age between the two groups, with younger patients showing a more altered HRCT. It is likely due to differences in the severity of the underlying disease. Patients in the study had different forms of immunodeficiencies, with heterogeneous clinical presentations. Indeed, most patients with altered chest HRCT have a more severe disease expression, with an earlier onset of symptoms, while patients with normal HRCT generally have a less severe disease expression and a milder severity of the symptoms on the whole.

Another limitation of the study is represented by the length of the observational period. Accordingly, a follow-up of 4 years may still be temporarily limited to catch clinically relevant radiological changes. Furthermore, four patients were enrolled during the study just at the initial phase of their follow-up. In any case, the relative stability of their clinical picture may support the validity of the surveillance and the personalized respiratory physiotherapy adopted.

Carrying out PFTs and HRCT on the same day was hard to organize for practical reasons. Therefore, we had to account for the time between HRCT and PFT for each patient, at the baseline and at the time of follow-up, respectively. However, the assessments were always performed in wellbeing states, and the statistical analysis did not demonstrate the influence on radiological results of the between-date intervals.

We conducted a pilot study on MBW in patients with PADs. In our sample, LCI appears as a parameter with higher sensitivity to predict the presence of structural abnormalities detectable by chest HRCT than reduced FEV1. Our results are consistent with the literature on other chronic lung diseases (33–35), but they need further confirmation in studies with larger sample sizes. To observe a difference of 0.5 between the LCI mean of the two groups, assuming a standard deviation (SD) of 0.75 (37), we needed at least 37 patients in each group.

Further studies on larger cohorts of pediatric patients with PADs followed longitudinally up to adulthood within a multicentric context are required to validate the usefulness of MBW in this population. An adequate sample size would also allow for a comparison between subjects with the same type of human inborn error of immunity (considering the different forms of PADs) and a consequent more detailed analysis of the different subgroups of patients, according to the emerging concepts of precision medicine.

## Data Availability Statement

The raw data supporting the conclusions of this article will be made available by the authors, without undue reservation.

## Ethics Statement

The studies involving human participants were reviewed and approved by IRCCS Fondazione Ca' Granda, Milan, Italy. Written informed consent to participate in this study was provided by the participants' legal guardian/next of kin.

## Author Contributions

TS, LB, and AL: full access to all the data in the study and take responsibility for the integrity of the data and the accuracy of the analysis. RD, LB, CA, and MA: concept and design. TS, LB, RD, AL, and CA: drafting of the manuscript and critical revision of the manuscript for important intellectual content. AL: statistical analysis. TS, LB, CA, and RD: administrative, technical, or material support. All the authors: acquisition, analysis, or interpretation of data. All authors contributed to the article and approved the submitted version.

## Funding

The study was partly supported by funding from the Italian Ministry of Health for IRCCS Institutes.

## Conflict of Interest

The authors declare that the research was conducted in the absence of any commercial or financial relationships that could be construed as a potential conflict of interest.

## Publisher's Note

All claims expressed in this article are solely those of the authors and do not necessarily represent those of their affiliated organizations, or those of the publisher, the editors and the reviewers. Any product that may be evaluated in this article, or claim that may be made by its manufacturer, is not guaranteed or endorsed by the publisher.

## Abbreviations

A-CT: altered HRCT
AIEOP: Italian Association of Pediatric Hematology and Oncology
ARA: autosomal recessive agammaglobulinemia
CF: cystic fibrosis
CI: confidence interval
CVID: common variable immunodeficiency
EC: Ethics Committee
ESID: European Society for Immunodeficiencies
FEV1: forced expiratory volume at 1 s
HIGM: hyper IgM syndrome
HRCT: high resolution computed tomography
Ig: immunoglobulin
IPINet: Italian Primary Immunodeficiencies Network
IQR: interquartile range
LCI: lung clearance index
LLN: lower limit of normal
MBW: multiple breath washout
MR: moment ratio
N-CT: normal HRCT
OR: odds ratio
PADs: primary antibody deficiencies
PFTs: pulmonary function tests
SD: standard deviation
SIgAD: selective IgA deficiency
ULN: upper limit of normal
XLA: X-linked agammaglobulinemia.
