# Supplementary material for: Multiple Breath Washout for Early Assessment of Pulmonary Complications in Patients With Primary Antibody Deficiencies: An Observational Study in Pediatric Age
Source: Front Pediatr. 2022 May 17;10:773751. doi: 10.3389/fped.2022.773751 (PMC9152221; doi:10.3389/fped.2022.773751)
Supplement: Supplementary file 1 [file Table_1.DOCX]

**Supplementary Table 1:** ORs, CIs. and p-values of univariate and multivariate (age and gender-adjusted) logistic regression analyses using standardized FEV1 and LCI as independent variables and A-TC/N-TC or Bhalla score under 3 (B0-2)/ Bhalla scores equal or more than 3 (B>2) as dependent variables. We performed the analysis at time 0 (T0) and after four years (T1). The CIs are calculated using the bootstrap method.

| ***Logistic regression*** | ***OR*** | ***CI*** | ***p-value*** |
| --- | --- | --- | --- |
| *Univariate FEV1 and A-TC/N-TC at T0* | 0,6 | 0,2 - 2 | 0.4 |
| *Univariate LCI and A-TC/N-TC at T0* | 2.3 | 0.1 - 52 | 0.4 |
| *Univariate FEV1 and A-TC/N-TC at T1* | 1.6 | 0.1 - 13.6 | 0.4 |
| *Univariate LCI1 and A-TC/N-TC at T1* | 3.9 | 0.2 -191 | 0.2 |
| *Adjusted FEV1 and A-TC/N-TC at T0* | 0.8 | 0 - 1.8e+43 | 0.8 |
| *Adjusted LCI1 and A-TC/N-TC at T0* | 2.4 | 0 - 7.6e+70 | 0.4 |
| *Adjusted FEV1 and A-TC/N-TC at T1* | 1.9 | 9.3e+44 | 0.4 |
| *Adjusted LCI1 and A-TC/N-TC at T1* | 14.9 | 0 – 1.9e+60 | 0.1 |
| *Univariate FEV1 and B0-2/B>2 at T0* | 0.5 | 0 – 9.3 | 0.3 |
| *Univariate LCI1 and B0-2/B>2 at T0* | 3.3 | 0 - 304567136 | 0.1 |
| *Univariate FEV1 and B0-2/B>2 at T1* | 0.5 | 0 – 2.8 | 0.4 |
| *Univariate LCI1 and B0-2/B>2 at T1* | 117.9 | 0 – 5.9e+65 | 0.3 |
| A-CT: Altered HRCT  B0-2: Bhalla score 0 -2  B>2: Bhala score more than 2  CI: Confidence Interval  FEV1: Forced Expiratory Volume at 1 second (standardized)  LCI: Lung Clearance Index (standardized)  N-CT: Normal HRCT  OR: Odds Ratio  T0: time 0  T1: time 1 (after 4 years) | | | |
